# Multiple Dimensions of Self-Esteem and Their Relationship with Health in Adolescence

**DOI:** 10.3390/ijerph17082616

**Published:** 2020-04-11

**Authors:** Francesca Pazzaglia, Angelica Moè, Sabrina Cipolletta, Monica Chia, Paola Galozzi, Stefano Masiero, Leonardo Punzi

**Affiliations:** 1 Department of General Psychology, University of Padova, 35131 Padova, Italy; francesca.pazzaglia@unipd.it (F.P.); sabrina.cipolletta@unipd.it (S.C.); 2 Health and Motion Venice Association (HEMOVE), 30122 Venice, Italy; monica.chia@virgilio.it (M.C.); paola.galozzi@unipd.it (P.G.); leonardo.punzi@unipd.it (L.P.); 3 Department of Medicine DIMED, University of Padova, 35128 Padova, Italy; 4 Department of Neuroscience, Rehabilitation Unit, University of Padova, 35128 Padova, Italy; stef.masiero@unipd.it; 5 Center for Gout and Metabolic Bone and Joint Diseases, Rheumatology, SS Giovanni and Paolo Hospital, 30122 Venice, Italy

**Keywords:** self-esteem, adolescence, lower back pain, physical exercise, smoking, health, wellbeing

## Abstract

The aim of the present study was to examine how different domains of self-esteem (social, competence, affect, academic, family, and physical) relate to self-reported physical and mental health, lower back pain (LBP), smoking, and physical exercise in a sample of adolescents. A sample of 326 adolescents 14–19 years old completed several self-report questionnaires collecting epidemiological data, and information on their LBP, smoking, and physical exercise, the Short Form Health Survey (SF-36), and the Multidimensional Self-Concept Scale. Pearson’s correlations were calculated between their self-esteem scores and their physical and mental health scores. Three multivariate analyses of variance (MANOVAs) were performed to estimate associations between self-esteem and LBP, smoking, and physical exercise. Self-esteem (total and subcomponent scores) correlated positively with physical and mental health, and with physical exercise, and negatively with smoking. The results also confirm gender-related differences in self-esteem, in favor of boys. This study offers the first findings on the relationship between different domains of self-esteem and a variety of health outcomes in an adolescent population. The results suggest that multidimensional interventions could be devised to improve adolescents’ physical health by promoting their physical exercise, and to prevent their smoking by nurturing their self-esteem.

## 1. Introduction

Self-Esteem (SE) can be defined as “the positivity of the person’s self-evaluation” [1]. It influences the development of important life outcomes, such as satisfaction in relationships, education, job success, and mental and physical health [2]. SE is particularly low during adolescence, and even lower in girls than in boys [3]. This requires particular attention because a low SE in adolescence can affect a range of health outcomes at this age, and also have cumulative effects on subsequent life outcomes.

Most studies on this issue, except [4], considered a global SE measure, without assessing whether health outcomes related differently to different domains of SE (e.g., affect, academic, family, physical). The present study fills this gap by examining domain-specific SE in adolescent girls and boys, and how it correlates with a very common self-reported physical symptom (lower back pain), and two lifestyle factors, one healthy (physical exercise), the other unhealthy (smoking habit).

### 1.1. Lower Back Pain and Healthy Behaviors

Nonspecific (common) lower back pain (LBP) is defined as pain and discomfort localized below the costal margin and above the inferior gluteal folds, with or without leg pain, not attributable to any recognizable, known specific disorders. It is a common condition in the industrialized world, and a relevant issue for national health services [5,6]. LBP is a sensory and emotional experience that has intense effects on well-being and is often cause of significant physical and psychological disability. Available literature indicates a clear link between psychological variables and back pain, and shows that distress, anxiety, negative mood and emotions are all significant factors, often related to the onset of pain, and to acute, subacute, and chronic pain [7,8,9]. Psychological factors (notably distress, depressive mood, and somatization) are also implicated in the transition to chronic low back pain and SE in adult patients.

Psychological factors are even known to play a significant role in the development of LBP, and previous mental disorders are positively associated with subsequent pain onset, and with patient outcomes [10,11,12,13,14]. Hence, the importance of considering this disease from a biopsychosocial perspective [10,12]. Assessing LBP consequently also involves considering variables such as the type of pain experienced, functional limitations, coping strategies, fear avoidance, and SE [13].

LBP is becoming as common in adolescents as in adults: its prevalence in adolescent age (up to 51%) is rising strongly enough to approach that of adults [15]. In spite of this, the main part of the studies on LBP are mainly focused on adults, and they analyzed SE in very specific terms, as SE in the ability to perform certain type of physical activity, such as lifting loads. In adolescents the relationship between SE and LBP is understudied, and no studies have so far explored the relationship between specific domains of SE and LBP. Hence, having more knowledge on such a connection could be useful in implementing interventions that take in account both physical and psychological correlates, in a critic age when the disease has an onset.

Smoking and physical exercise have been widely recognized as key health behaviors [8]: nearly one in two deaths in the United States are caused by behavioral factors, with smoking and inactivity among the main reasons. Smokers report major limitations with daily life, and consistently poorer health and less perceived wellbeing than non-smokers [9]. SE has been found to be positively associated with past and present smoking, and with excessive alcohol consumption [16]. The habit of smoking is often acquired already in late childhood or early adolescence. It can be predicted by variables such as: increasing age or school grade; weak academic performance; lower socio-economic status; sensation seeking or rebelliousness; intention to smoke in the future; receptivity to tobacco promotion efforts; susceptibility to smoking; having family members who smoke; having friends who smoke; and exposure to movies. On the other hand, a higher SE and close parental monitoring seem to protect young people against developing a smoking habit [17]. In general, apart few exceptions, the studies that analyzed the relationship between smoking behavior and a general measure of SE found an association. Associations with specific dimensions of SE were also found. SE for physical appearance was found to mediate the effect of body mass index on smoking. However, only few studies analyzed a wide range of SE dimensions, with inconsistent results, so the relation between smoking and SE should be further analyzed.

Moreover, participating in sports (egalitarian, elite, and entertainment sports) favor mental health [18]. Apart from the physical exercise aspect of practicing a sport, which is known to have positive effects on mental health, the most prevalent effects of sport lie in expanding and strengthening social networks, a variable that is associated with a better perceived wellbeing [19].

### 1.2. Self-Esteem and Health

Many studies confirmed that SE supports health generally, both mental and physical [20], and vice versa [21]. Since physical health tends to decline in old age, most of these studies focused on adults or the elderly [22], and on single health problems, such as hypertension or cancer [23]. Only a few considered particular physical symptoms [24] and none—to the best of our knowledge—investigated adolescents.

Adolescent SE has been shown to have a bearing on depression [25] and physical health [26] later in life, so it can have far-reaching effects. Physical symptoms reported during adolescence may correlate with an individual’s SE, and therefore influence their SE and health as an adult too.

SE has revealed a link not only with health, but also with how people cope with health problems or physical symptoms [27]. The mechanism behind this association lies in that people with high levels of SE being more likely to request and obtain social support and experience less helplessness and stress. This prompts them to adopt more effective coping modalities, which in turn contributes to solving their health problems and containing their physical symptoms [28].

### 1.3. Assessing Self-Esteem in Adolescence

SE is frequently assessed with a 10-item self-report instruments to obtain a single dimension referring to a global measure of an individual’s self-worth [29]. Later on, a two-dimensional model based on two core dimensions, self-liking (mostly emotional in nature) and self-competence (mostly cognitive) was developed [30], together with a self-testing instrument for measuring them. These two dimensions are independent, but inter-related: the more we see ourselves as being competent, the better we like ourselves.

These measures are typically used with adults, including the elderly. For children and adolescents, whose SE is still changing and growing [31], an instrument called the Multidimensional Self-Concept Scale (MSCS) [32] was developed. The scale considers six domains (social, competence, affect, academic, family, and physical), each of which is assessed by means of 25 items. Given our interest in adolescence, and the speculation that specific domains (e.g., social, affect, or physical) might correlate more strongly with health (as found with smoking habits: [33,34]), we adopted the multi-dimensional approach and the MSCS, in its Italian validation [35].

### 1.4. Gender-Related Differences in Self-Esteem

SE increases from adolescence to midlife in both genders, but girls tend to have a lower SE than boys for decades [2,3]. This gender-related difference in SE is generally small in size [36,37]: a meta-analysis found an overall Cohen’s *d* of 0.21 [38]. This gap is wider in adolescence (age 15–18 years), however, than at any other age (*d* = 0.33), as recently confirmed [39], who emphasized the need to consider adolescents and the consequences of their lower SE more carefully.

Looking at single domains instead of an overall measure of SE, a medium-sized male advantage in SE for the physical appearance, athletic, personal self, and self-satisfaction domains was found [4]. Females showed a medium-sized advantage for moral-ethical SE, and a small one for behavioral conduct. There were no differences in SE by gender for the academic, family, and social acceptance domains. A further study [40] confirmed that girls had a lower SE than boys regarding physical appearance, but also found evidence of lower social and academic SE in girls. While these results indicate that gender-related differences in SE can disappear or even be reversed in certain domains, depending on the study, they nonetheless confirm that girls and women have consistently lower SE than boys and men as regards their physical appearance.

### 1.5. Aims and Hypothesis

This study aims to explore the relationship between different dimensions of SE (social, competence, affect, academic, family, and physical) and self-reported physical and mental health in a sample of adolescents. It also examines how SE dimensions relate to self-reported physical symptoms of LBP, and to healthy and unhealthy lifestyle factors (regular physical exercise and smoking habit, respectively). Gender-related differences in SE are examined as well.

On the strength of past evidence in adult participants, we expected to find associations between their SE and their physical and psychological health, in a sample of adolescents. We expected SE to be higher in boys than in girls, both for the physical subcomponent as in [4,40], and as a whole, as found by [35] on a sample of 1062 Italian 12- to 15-year-old boys and girls). In our group of adolescents, we also newly explore how SE, considered as a global measure and in its sub-components, correlates with LBP: we expected adolescents who reported having suffered from LBP to score lower on SE than those who had not. We also predicted that SE would be negatively associated with smoking habit, and positively associated with regular physical exercise.

## 2. Method

### 2.1. Participants

The study sample included a total of 326 students aged 14–19 years (mean = 16.89, SD = 0.93), attending the 2nd to 4th years at various secondary schools in Venice, Verona, and Padua (Italy). Schools and students participated in the project on a voluntary basis, following the decision of the management teams of some secondary schools of Padua, Venice, and Verona to participate in a project with the University Hospital of Padua. The whole project was sponsored by the Italian Ministry of Education, University and Research (MIUR) and regulated by the Alternation School-Work Law 107 of 13 July 2015. The study was conducted in accordance with the Declaration of Helsinki, and the protocol was approved by the Ethics Committee of Department of Biomedical Sciences (Project identification code HEC-DSB/02-19).

### 2.2. Measures

**Epidemiological questionnaire.** We used a non-validated, structured self-reporting questionnaire designed for a wider survey to collect epidemiological data and information on any presence and characteristics of LBP, and to quantify its impact on health status and health-related quality of life. The questionnaire is divided into sections. The first part is for personal details (e.g., date of birth, gender, residence, school attended and year, physical exercise, musical instruments played, smoking) and anthropometric data (height, weight). The second part includes questions on any past and present LBP, and treatments, instrumental analyses, etc. (beyond the scope of the present study). The last part contains questions about the consequences of LBP on social functioning (absences from school, interference with daily physical activities).

This epidemiological questionnaire was devised for broader epidemiological purposes than those of the present study [15], so we only examined part of the numerous variables envisaged (those more pertinent to the goals of the present study), i.e., gender, regular physical exercise (yes/no), smoking habit (yes/no), back pain in the past (yes/no).

**Quality of Life Questionnaire.** To assess health-related quality of life we adopted the Italian version of the Short Form Health Survey (SF-36) questionnaire [41], which is a reliable and valid instrument that measures two major health concepts (physical and mental health) with 36 items, and generates eight multi-item scales: physical functioning (PF), physical role limitation (RP), bodily pain (BP), general health (GH), vitality (VT), social functioning (SF), emotional role limitation (RE), and mental health (MH). The sum of each respondent’s scores was calculated, then the raw scores were linearly transformed into 0–100 scales, with 0 and 100 assigned respectively to the lowest and highest possible values. Higher scores indicated a better physical and mental functioning. For the purposes of the present study, we only considered the variables GH and MH, Cronbach alphas of 0.77 and 0.85, respectively.

**Self-esteem questionnaire.** The Multidimensional Self-Concept Scale [32] was used in its Italian validation [35]. This scale comprises 150 items evenly distributed to obtain six subscales (with 25 items each) assessing different SE domains: social, competence, affect, academic, family, and physical. Cronbach’s alphas calculated on the present sample for the whole scale and each subscale were always ≥84.

### 2.3. Procedure

The questionnaires were administered in class during school hours by two members of the research team. Specific instructions were given before administering each questionnaire, then students completed them on printed paper copies. The time for data collection was decided together with a representative of the teachers of the schools involved during preliminary meeting where goals and procedure were accurately explained. On the appointed day, the questionnaires were administered at the same time to all the volunteer students in a large classroom inside the school building. The students were from different classrooms and received an envelope containing the three questionnaires (in random order) and relative instructions. They were invited to sit down and fill in the questionnaires. They were allowed to eventually ask the experimenters for clarifications when necessary and no time limits were given. The questionnaires were completed at different times for each school, but all the students from a school were in the same classroom at the administration.

### 2.4. Data Analysis

Pearson’s correlations between the scores for the six MSCS domains and the SF-36 total scores for SE, and for physical and mental health, were calculated on the sample as a whole, and separately by gender. The gender-related differences in SE and health scores were examined with a series of t-tests. Three multivariate analyses of variance (MANOVAs) were run on the six MSCS subscales and the total SE scores, with LBP, smoking habit, or physical exercise as the only factor.

## 3. Results

### 3.1. Correlations Between Self-Esteem (SE) and Short Form Health Survey (SF-36) Indexes

Correlations are shown in Table 1 (for the whole sample) and Table 2 (by gender). Descriptive statistics for each of the variables considered are also shown, by gender, in Table 2. For both the whole sample, and the two subsamples split by gender, the correlations between SE scores and physical and psychological health (measured by the SF-36) are moderate and significant.

In the sample as a whole, the correlations are stronger for competence, affect, and physical SE, than for the social, academic, and family domains. The correlations are generally stronger for girls than for boys, and—in the sample as a whole—they suggest a close relationship between SE and health.

### 3.2. Gender Differences

Table 2 shows the mean values and the comparisons drawn with Student’s t-test together with the R^2^. Gender-related differences emerged for: physical and mental health, and SE (total scores, and scores for the social, affect, and physical domains). The difference in SE for competence was marginally significant (*p* = 0.05). In all these differences, boys obtained higher SE scores than girls. The percentage of variance explained by gender was higher for SE-physical (0.09), followed by mental health and SE-affect (0.06 and 0.05, respectively). Regarding SE-total, SE-social, and physical health, the factor gender explained 4% of variance, whereas the effect was very low for SE-academic, SE-competence, and SE-family.

### 3.3. Differences in SE Relating to Lower Back Pain (LBP), Smoking Habit, and Physical Exercise

To further examine the relationship between SE, health, and behavior, we compared the SE scores of participants who reportedly had or had not suffered from LBP, did or did not smoke, and did or did not engage in regular physical exercise.

In a series of preliminary analyses, gender did not enter into any interaction with LBP, smoking habit, or physical exercise, so we performed three MANOVAs with LBP, smoking or physical exercise as the only factor. Mean scores and standard deviations for the SE total score and subscales are shown in Table 3, distinguishing between: (1) LBP sufferers versus others; (2) smokers vs. non-smokers; and (3) physical exercise practitioners vs. others.

The MANOVAs suggested that LBP, smoking, and physical exercise each have an effect at multivariate level on SE [LBP: *F*(6,319) = 3.62, *p* = 0.002, Pillai’s trace = 0.06; SMOKING: *F*(6,315) = 3.93, *p* < 0.001, Pillai’s trace = 0.07; PHYSICAL EXERCISE: *F*(6,319) = 3.21, *p* < 0.001, Pillai’s trace = 0.01. As seen in Table 4, participants who suffered from LBP had lower scores for total SE, and all the single domains except academic, than participants who did not. Smokers had scored lower than no-smokers for total SE, and in the social, affect and academic domains. Participants who reported engaging in physical exercise scored higher than those who did not, with differences in total SE, and in the social, affect, and physical domains.

Finally, we performed a t-test analysis to compare SE-total of LBP sufferers who declared that LBP limited their every-day physical activity with those who did not. The difference between the two groups was significant, *t* (256) = 2.35, *p* = 0.19, R^2^ = 02, with lower associated to the feeling of being limited (Limited: *M* = 2.77, *SD* = 0.34; not-limited: *M* = 2.87, *SD* = 0.30).

## 4. Discussion

LBP is a common condition in industrialized societies, and its incidence is rising in adolescents [15]. Psychological factors, including depression, pain catastrophizing, fear and avoidance, are linked to poor outcomes in low back pain [12]. The adoption of a healthy (e.g., physical exercise) or unhealthy (e.g., smoking) lifestyle is frequently associated with psychological factors [10,11,12,13,14]. One of the psychological factors most relevant in influencing mental and physical health is SE [2]. SE is low during adolescence [3], a potentially crucial time of life for the onset of dysfunctional associations between a low SE, poor physical and mental health, and unhealthy behavior, such as smoking. Hence this study to examine the relations between SE, self-reported LBP, and unhealthy and healthy behaviors (smoking and physical exercise, respectively) in adolescence. SE was examined as a multidimensional construct [32], obtaining a total measure of SE resulting from its assessment in the social, competence, affect, academic, family, and physical domains. It is important to consider such SE sub-components separately because some of them (e.g., social and physical SE) might be particularly relevant in adolescence, and more closely related to an individual’s physical and mental health.

The main findings of our study confirm the relationship between SE and physical and mental health. The measures of physical and mental health derived from the SF-36 questionnaire correlated with the scores for total SE and all the SE sub-components, confirming that the association between SE and health already described in adults [2] applies to adolescents too. A link was also found when we considered a specific health issue (LBP), and healthy or unhealthy lifestyle factors. Adolescents who reported having suffered from LBP scored lower for total SE, and in all the single domains except academic, than those who had never reportedly suffered from LBP. The effect sizes were overall small and in the range 0.20 to 0.50 as can be seen in Table 4 [42]. Only for physical SE as about the healthy attitude towards physical exercise the Cohen d was up to 0.50, equal to 0.58. showing and important effect of physical exercise on this sub-dimension of self-esteem. Even if not large, these differences are all relevant and in the expected direction showing a small, but interesting, effect of not smoking, nor suffering from LBP and doing physical exercise on SE. Moreover, being the effects slightly higher for physical exercise than for an unhealthy habit (smoking) or a physical complain (back pain) suggest that acting on fostering positive attitudes and lifestyles can probably raise self-esteem more than acting on reducing unhealthy habits or attitudes.

Moreover, among LBP sufferers, those who declared to be limited in every-day activities had total-SE lower than those who did not. This is a novel result because no previous study explored this relationship in an adolescent population. Moreover, this data suggests a useful direction for future studies, which might focus on a specific health dimension rather than analyzing the effects of some health behaviors and psychological characteristics on general indicators of wellbeing or quality of life.

In line with previous studies [17], SE revealed correlations both with an unhealthy type of behavior, like smoking (with smokers scoring lower for SE), and with a healthy one such as engaging in physical exercise (which was associated with a higher SE). These relationships can be explained in various ways, and further research will be needed to clarify the causal relations and specific mechanisms involved. A low SE may be associated with less functional coping strategies [43], such as drinking alcohol, over-eating, smoking, and social withdrawal, with a negative impact on physical health. On the other hand, poor health could have negative consequences on sense of competence, interpersonal relations, and physical exercise, with detrimental effects on SE. It is important to note that, whatever the direction of such causal relationships, there is probably a vicious circle at play involving with a low SE and the perception of poor physical and mental health.

As a secondary goal, we also examined gender-related differences in SE. Our results confirmed that girls report a lower SE than boys overall [38]. The effect of gender is higher in two dimensions (physical and affect), in which the explained variance was 0.09 and 0.05, respectively, while the difference is not significant for the SE-family dimension. This is in line with previous research results, mainly as about the physical SE where girls substantially differ from boys. The occurrence of large to null gender differences in subdimensions of self-esteem emphasizes the importance to deepen in future studies the knowledge of the factors leading girls to underestimation in particular in these domains. The literature examined the impact of role models leading to expectations focused on pointing at the ‘need’ for girls to be beauty and appear good [4]. However, future studies should consider also other factors, such as physical and mental health because the correlations with these dimensions and the physical-SE are higher for girls than boys, as shown in Table 2. Finally, this apparently greater vulnerability of girls to a low SE in specific domains should be taken into account when devising training to nurture adolescents’ SE.

### 4.1. Practical Implications

Our results suggest that education programs aiming to improve adolescents’ physical health should be accompanied by intervention on their SE and other psychological factors, such as coping strategies. For instance, action to prevention and reduce LBP should include training to nurture SE, especially for girls, as well as information about the physical causes of LBP and appropriate exercises. As concerns adolescents’ psychological wellbeing, it would seem that intervention on this sphere should not be separated from action focusing on physical health and healthy lifestyle choices. Since a good degree of SE in adolescence favors adult mental health [6], it is important to take steps to foster SE from adolescence onwards. Various methods have been proposed, such as encouraging realistic self-perceptions, increasing awareness of the origins of negative self-perceptions, and encouraging individuals to voice their social support needs [27]. SE interventions should also consider the various sub-components of this construct because some of them could be more important than others in a given individual. Importantly, in the present paper we studied the relationship between SE and LBP in an educational context. Our results should be tested also in clinical settings, in order to explore SE of adolescents suffering from severe LBP disease and the utility of a psychological support in physical rehabilitation trainings.

### 4.2. Limitations and Future Avenues

The present study has some limitations. First, it is based entirely on self-reports, and we cannot say whether the relations identified are objectively well-founded or merely based on participants’ perceptions. Future studies on smaller samples with objective medical reports could be more informative regarding such relations. It is important to bear in mind, however, that perceptions of mental and physical health are sometimes more influential than self-representations or actual conditions. Second, this was a cross-sectional study, necessarily based on correlations found at the time of data collection. This prevents us from drawing conclusions on the causal links between variables—something that only longitudinal studies, or studies based on training programs, could achieve. In this respect, it would be interesting to see whether a training program focusing only on SE could produce positive effects on mental and physical health. A third limitation concerns the fact that our sample consisted of an uneven number of boys and girls. Exploring gender-related differences was only a secondary goal of the study, however. It is also worth adding that the pattern of relations between SE and health was very similar in girls and boys, and gender did not enter into any of the interactions, suggesting that the associations found between SE and LBP, smoking, or physical exercise were unaffected by gender. Another limit is that our sample was recruited in the schools and we could not collect data from adolescents who dropped out from the education system (about 10% of population in the area of Italy involved; [44]). Moreover, given that being NEET (Not in Education, Employment, or Training) is associated with negative physical and psychological factors [45], future research should consider recruiting participants in non-school contexts to confirm and extend the results here obtained.

## 5. Conclusions

Single SE domains correlated with LBP and the adoption of unhealthy or healthy behaviors in a sample of adolescents—an age group whose SE levels could affect their wellbeing and health in later life. In conclusion, this study has the value of underscoring the link between SE and measures of mental and physical health in adolescents and suggesting directions for efforts to improve their physical and mental health.

## Figures and Tables

**Table 1:** Descriptive statistics and correlation coefficients (whole sample).

Variable	*M (SD)*	1	2	3	4	5	6	7	8
1. SE-total	2.85 (0.33)	/							
2. SE-social	2.93 (0.38)	0.76	/						
3. SE-competence	2.82 (0.36)	0.87	0.60	/					
4. SE-affect	2.66 (0.49)	0.89	0.67	0.75	/				
5. SE-academic	2.76 (0.33)	0.62	0.29	0.65	0.43	/			
6. SE-family	3.29 (0.49)	0.70	0.42	0.52	0.52	0.31	/		
7. SE-physical	2.65 (0.45)	0.83	0.61	0.66	0.75	0.42	0.42	/	
8. Physical health	69.61 (18.66)	0.46	0.28	0.39	0.48	0.26	0.23	0.49	/
9. Mental health	46.97 (20.94)	0.56	0.44	0.42	0.66	0.30	0.33	0.42	0.42

Note. SE = Self-Efficacy. All rs significant at *p* < 0.01.

**Table 2:** Descriptive statistics by gender, *t*-value (*df* = 323 for all variables except physical and mental health: *df* = 319), and correlation coefficients by gender (girls above the diagonal). SE = Self-Efficacy.

Variable	*Girls*	*Boys*											
	*M (SD)*	*M (SD)*	*t*	R^2^	1	2	3	4	5	6	7	8	9
1. SE-total	2.81 (0.32)	2.96 (0.32)	3.86 **	0.04	/	0.77 **	0.86 **	0.90 **	0.57 **	0.68 **	0.83 **	0.50 **	0.56 **
2. SE-social	2.88 (0.39)	3.05 (0.33)	3.68 **	0.04	0.68 **	/	0.59 **	0.72 **	0.25 **	0.40 **	0.61 **	0.31 **	0.51 **
3. SE-competence	2.80 (0.35)	2.88 (0.36)	1.96	0.01	0.91 **	0.60 **	/	0.75 **	0.63 **	0.47 **	0.65 **	0.42 **	0.42 **
4. SE-affect	2.59 (0.47)	2.84 (0.49)	4.11 **	0.05	0.86 **	0.46 **	0.75 **	/	0.36 **	0.49 **	0.78 **	0.53 **	0.63 **
5. SE-academic	2.74 (0.31)	2.81 (0.35)	1.60	0.01	0.73 **	0.34 **	0.68 **	0.57 **	/	0.24 **	0.38 **	0.25 **	0.24 **
6. SE-family	3.26 (0.48)	3.32 (0.50)	0.89	0.00	0.79 **	0.46 **	0.64 **	0.60 **	0.45 **	/	0.40 **	0.27 **	0.35 **
7. SE-physical	2.57 (0.44)	2.87 (0.39)	5.56 **	0.09	0.79 **	0.52 **	0.69 **	0.64 **	0.48 **	0.48 **	/	0.51 **	0.40 **
8. Physical health	67.29 (19.14)	75.38 (15.89)	3.55 **	0.04	0.22 *	0.03	0.28 **	0.23 *	0.24 *	0.05	0.25 *	/	0.43 **
9. Mental health	44.48 (20.36)	53.37 (19.64)	3.54 **	0.06	0.47 **	0.13	0.39 **	0.66 **	0.39 **	0.29 **	0.32 **	0.28 **	/

Note: * *p* < 0.05; ** *p* < 0.01.

**Table 3:** Descriptive statistics for total SE scores and SE subscales by lower back pain (LBP) sufferers vs. non-sufferers, smokers vs. non-smokers, sports practitioners vs. non-practitioners. SE = Self-efficacy.

Variable	No LBP	LBP	Non-smokers	Smokers	No-Physical Exercise	Physical Exercise
	M (SD)	M (SD)	M (SD)	M (SD)	M (SD)	M (SD)
1. SE-total	2.91 (0.32)	2.80 (0.33)	2.87 (0.34)	2.77 (0.29)	2.78 (0.32)	2.90 (0.32)
2. SE-social	3.00 (0.32)	2.87 (0.42)	2.93 (0.38)	2.95 (0.39)	2.88 (0.39)	2.96 (0.37)
3. SE-competence	2.88 (0.35)	2.77 (0.35)	2.86 (0.36)	2.69 (29)	2.75 (0.36)	2.87 (0.34)
4. SE-affect	2.75 (0.50)	2.59 (0.47)	2.69 (0.50)	2.56 (0.45)	2.59 (0.49)	2.70 (0.48)
5. SE-academic	2.75 (0.34)	2.77 (0.32)	2.79 (0.33)	2.66 (0.31)	2.72 (0.32)	2.79 (0.33)
6. SE-family	3.34 (0.45)	3.22 (0.52)	3.31 (0.48)	3.18 (0.52)	3.21 (0.52)	3.32 (0.46)
7. SE-physical	2.75 (0.44)	2.57 (0.45)	2.68 (0.46)	2.57 (43)	2.50 (0.45)	2.76 (0.43)

**Table 4:** Results of single ANOVAs from MANOVAs.

Variable		Total	Social	Competence	Affect	Academic	Family	Physical
LBP	*F*	9.49	8.99	6.54	8.98	0.28	5.19	12.17
	*p*	0.002	0.003	0.011	0.003	ns	0.023	0.001
	*MSE*	0.06	0.14	0.12	0.23	0.11	0.24	0.20
	*Cohen’s d*	0.34	0.33	0.28	0.33	−0.06	0.25	0.39
Smoking	*F*	5.76	0.24	12.08	4.07	P 8.68	3.36	3.45
	*p*	0.017	ns	0.001	0.044	0.003	ns	ns
	*MSE*	0.11	0.15	0.12	0.24	0.10	0.24	0.21
	*Cohen’s d*	0.33	−0.07	0.47	0.27	0.40	0.25	0.25
Physical exercise	*F*	11.47	3.17	9.15	4.18	3.74	3.55	26.73
	*p*	0.001	ns	0.003	0.042	0.05	ns	<0.001
	*MSE*	0.10	0.14	0.12	0.24	0.11	0.24	0.19
	*Cohen’s d*	−0.38	−0.20	−0.34	−0.23	−0.22	−0.21	−0.58
